# Duodenal microbiota dysbiosis in functional dyspepsia and its potential role of the duodenal microbiota in gut–brain axis interaction: a systematic review

**DOI:** 10.3389/fmicb.2024.1409280

**Published:** 2024-08-06

**Authors:** Xueping Zhang, Lei Chen, Tao Zhang, Ryu Gabo, Qianying Wang, Zhuotai Zhong, Mengxi Yao, Wei Wei, Xiaolan Su

**Affiliations:** Department of Gastroenterology, Beijing Key Laboratory of Functional Gastrointestinal Disorders Diagnosis and Treatment of Traditional Chinese Medicine, Wangjing Hospital, China Academy of Chinese Medical Sciences, Beijing, China

**Keywords:** microbiota, functional dyspepsia, duodenum, systematic review, gut-brain interaction

## Abstract

**Background and aims:**

Functional dyspepsia (FD) is a common gastrointestinal disorder associated with brain–gut interaction disturbances. In recent years, accumulating evidence points to the duodenum as a key integrator in dyspepsia symptom generation. Investigations into the pathological changes in the duodenum of FD patients have begun to focus on the role of duodenal microbiota dysbiosis. This review summarizes duodenal microbiota changes in FD patients and explores their relationship with gut-brain interaction dysregulation.

**Methods:**

Ten databases, including PubMed, MEDLINE, and the Cochrane Library, were searched from inception to 10th October 2023 for clinical interventional and observational studies comparing the duodenal microbiota of FD patients with controls. We extracted and qualitatively summarized the alpha diversity, beta diversity, microbiota composition, and dysbiosis-related factors.

**Results:**

A total of nine studies, consisting of 391 FD patients and 132 non-FD controls, were included. The findings reveal that the alpha diversity of the duodenal microbiota in FD patients does not exhibit a significant difference compared to non-FD controls, although an upward trend is observed. Furthermore, alterations in the duodenal microbiota of FD patients are associated with the symptom burden, which, in turn, impacts their quality of life. In FD patients, a considerable number of duodenal microbiota demonstrate a marked ascending trend in relative abundance, including taxa such as the phylum *Fusobacteria*, the genera *Alloprevotella*, *Corynebacterium*, *Peptostreptococcus*, *Staphylococcus*, *Clostridium,* and *Streptococcus*. A more pronounced declining trend is observed in the populations of the genera *Actinomyces*, *Gemella*, *Haemophilus*, *Megasphaera*, *Mogibacterium*, and *Selenomonas* within FD patients. A negative correlation in the relative abundance changes between *Streptococcus* and *Prevotella* is identified, which correlates with the severity of symptom burden in FD patients. Moreover, the alterations in specific microbial communities in FD patients and their potential interactions with the gut–brain axis merit significant attention.

**Conclusion:**

Microbial dysbiosis in FD patients is linked to the onset and exacerbation of symptoms and is related to the disorder of gut–brain interaction. Larger-scale, higher-quality studies, along with comprehensive meta-omics research, are essential to further elucidate the characteristics of the duodenal microbiota in FD patients and its role in FD pathogenesis.

**Systematic review registration**: CRD42023470279, URL: https://www.crd.york.ac.uk/prospero/display_record.php?ID=CRD42023470279.

## 1 Introduction

Functional dyspepsia (FD) is one of the most prevalent functional gastrointestinal (GI) disorders of gastroduodenal origin (Stanghellini et al., 2016). The global prevalence of FD is approximately 16% and may vary substantially according to different countries and the definition criteria of the disease (Ford et al., 2020). FD is defined as the presence of one or more of four symptoms: postprandial fullness, early satiation, epigastric pain, and epigastric burning, and cannot be explained by structural or biochemical abnormalities identified in routine clinical settings (Drossman, 2016; Ford et al., 2020). Based on the predominant symptom pattern, Rome IV provides for two functional dyspepsia subtypes of FD: epigastric pain syndrome (EPS) with epigastric pain and/or epigastric burning and postprandial distress syndrome (PDS) with postprandial fullness and/or early satiety (Drossman, 2016). Although FD does not affect survival, the symptoms of FD can be quite troublesome and difficult to treat, and patients usually have a natural history of recurrence and remission. FD is believed to affect the diet (quantity and quality of meals) and quality of life of patients, reduce the productivity of patients, lead to emotional disorders and somatization, and may result in high medical costs, which seriously burden individuals and society (Lacy et al., 2013; Gracie et al., 2015; Aziz et al., 2018; Esterita et al., 2021).

Due to the multifactorial and heterogeneous nature of functional dyspepsia symptoms, many factors influence the pathogenesis of FD. At present, the mainstream theory believes that the key to the pathogenesis of FD is related to the disorder of gut–brain interaction (DGBI) (Zhou et al., 2022). In addition, emerging research in recent years has begun to point toward the duodenum as a key player in the pathogenesis of FD.

The gastric sensorimotor dysfunction observed in FD patients may be attributed to the activation of duodeno-gastric reflexes, which transmit noxious stimuli from the duodenal mucosa via afferent nerves, leading to abnormal gastric motility and hypersensitivity (Vanheel and Ricard, 2013; Miwa et al., 2019; Wauters et al., 2020a). As a pathogenic epicenter, the duodenum induces upper gastrointestinal symptoms in FD patients primarily due to the stimulation of duodenal contents, low-grade inflammation, and increased mucosal permeability in the duodenum. Alterations in the duodenal microbiota, or “dysbiosis,” are posited to be a significant factor contributing to the emergence of these duodenal pathologies (Miwa et al., 2019; Wauters et al., 2020a). Recent research has focused on the presence of small intestinal bacterial overgrowth (SIBO) in FD patients (Gurusamy et al., 2021a). Relevant studies have uncovered a dysbiotic state within the duodenal mucosa of FD patients, characterized by increased bacterial load and diversity. This microbial imbalance has been found to be associated with the manifestation of gastrointestinal symptoms in FD patients (Zhong et al., 2017). The gut microbiota may communicate with the central nervous system via neural, endocrine, and immune pathways, thereby influencing brain function. In FD patients, dysbiosis of the gut microbiota is known to affect the gut–brain interactive functions, with the microbiota-gut–brain axis being recognized as playing a significant role in FD (Rupp and Andreas, 2022).

However, in previous studies, due to the difficulty in obtaining duodenal microbiota samples, most studies on the gastrointestinal microbiota of FD patients focused on the microbiota of the stomach, large intestine, and feces; the understanding of duodenal microbiota was relatively limited (Tziatzios et al., 2020; Zhou et al., 2022). The dysbiosis of the duodenal microbiota and its implications for the pathogenesis of FD warrant further exploration.

Drawing from the aforementioned perspectives, we postulate that alterations in the duodenal microbiota represent a crucial yet underappreciated nexus influencing the pathogenesis of functional dyspepsia (FD) while also engaging in the gut–brain axis of FD patients. Recent observational studies have provided pertinent insights into the involvement of the duodenal microbiota in FD onset. Building upon these foundations, this study synthesizes the characteristic changes in duodenal microbiota among FD patients, endeavors to elucidate the mechanisms by which duodenal microbiota participate in gut–brain interactions, explores the role of duodenal microbiota alterations in FD pathogenesis, and endeavors to identify bacterial targets pertinent to FD diagnosis or treatment.

## 2 Methods

### 2.1 Protocol and registration

This systematic review followed the recommended approach described in the Preferred Reporting Items for Systematic Review and Meta-Analyses Protocols (PRISMA-P) 2015 Statement Guidelines (PRISMA-P Group et al., 2015; Page et al., 2020).

The protocol has been registered with the International Prospective Register of Systematic Reviews, and the PROSPERO registration number is CRD42023470279.

### 2.2 Search strategy

We performed a systematic search of 10 electronic databases, including PubMed, MEDLINE (Medical Literature Analysis and Retrieval System Online), Cochrane Library, ClinicalTrials.gov, Excerpta Medica Database (EMBASE), Web of Science (Wos), Wanfang Data, China National Knowledge Infrastructure (CNKI), VIP Information Resource Integration Service Platform (CQVIP), and Chinese Biomedical Literature Database (SinoMed), from inception to 10th October 2023, with the keywords “duodenum,” “microbiota,” and “functional dyspepsia.” We also manually searched for other relevant literature based on the references to the identified articles.

### 2.3 Study selection and patient population

This review selected observational and interventional studies (i.e., case–control, cohort, and randomized and non-randomized clinical trial studies) that compared the composition of the duodenal microbiota, microbial diversity, or microbial richness between FD patients and non-FD controls. FD patients must have a presumed diagnosis of FD based on the clinical assessment, questionnaire data, or specific symptom-based criteria, including the Rome criteria, and patients must not show any evidence of gastric/duodenal mucosal abnormalities, lesions, or structural changes (based on endoscopic and clinical histology findings). The control group (CG) comprises individuals without functional dyspepsia, including healthy controls, and is required to possess demographic data comparable to that of the FD group. There are no restrictions on the age and gender of the population. We excluded studies in which the experimental group included patients with functional gastrointestinal disorders or FD combined with other diseases. Case reports, expert opinions, and reviews were also excluded.

### 2.4 Data extraction

Data were extracted by two independent authors (XPZ and LC) and confirmed by a third (XLS). The following data were extracted: (1) study design, including the name of the first author, year of publication, journal, country, study design, inclusion and exclusion criteria, and criterion for FD diagnosis; (2) population characteristics, including age, sex, sample size, body mass index (BMI), follow-up duration, usage of proton pump inhibitor (PPI), and subtype(s) of FD if specified; (3) outcome measures: specimen processing (collection, storage, and DNA extraction), sequencing platforms, method of gut microbiota estimation, alpha diversity, beta diversity index, duodenal microbiota profile, and relative abundance. The primary outcome was the difference in individual duodenal bacterial species taxonomic classification reported in FD patients compared to non-FD controls.

### 2.5 Quality of studies

Two independent researchers (QYW and ZTZ) evaluated the risk of research bias separately. Study quality (risk of bias) has been assessed by the Newcastle-Ottawa scale (NOS) tool, which evaluates research through population selection, comparability, exposure, or outcomes (Stang, 2010). Any differences between the two researchers will be resolved through discussion or negotiation with the third reviewer (TZ).

## 3 Results

### 3.1 Study selection

**Figure 1:**
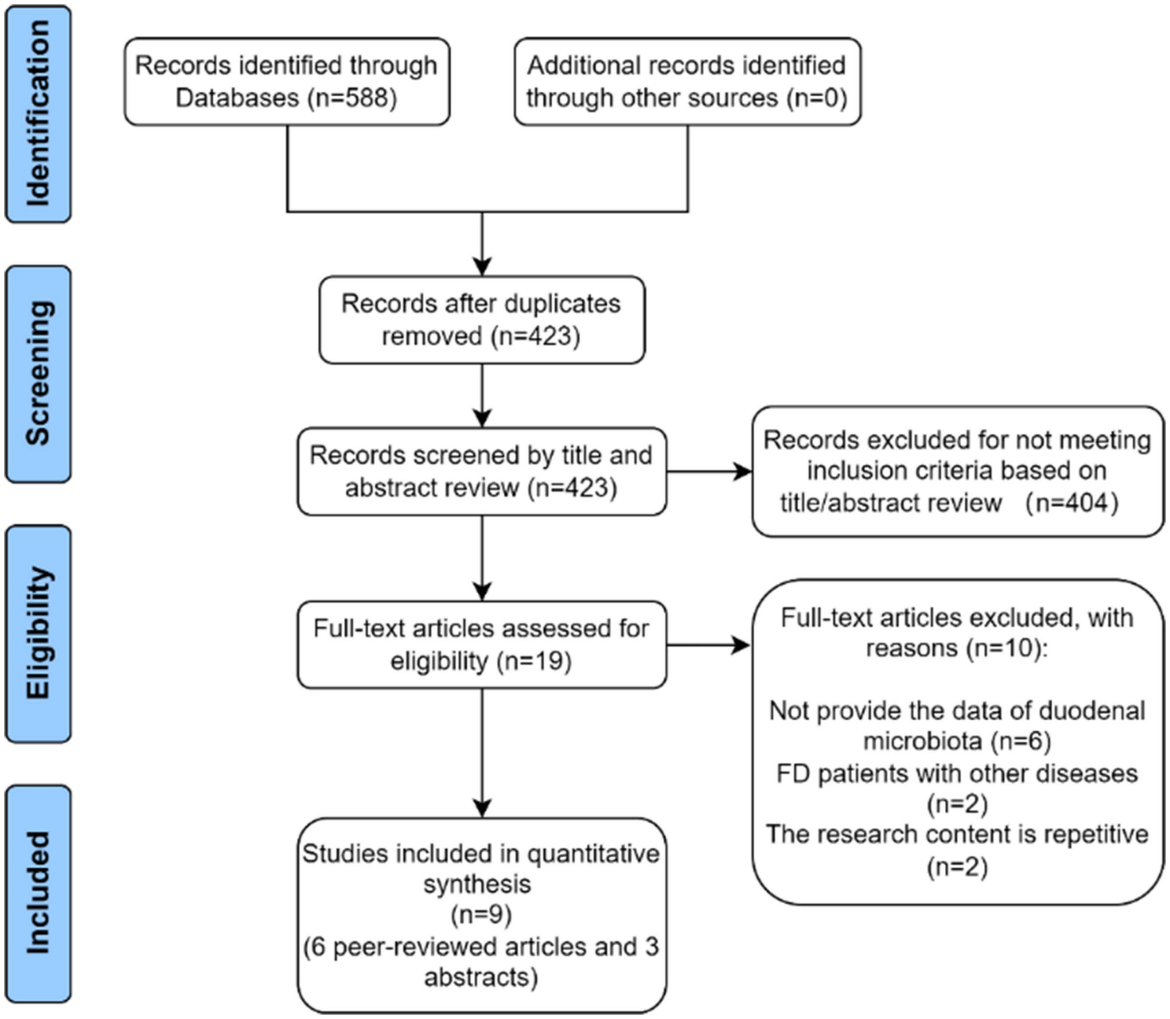
Flow diagram of study selection.

A total of 588 studies were identified in the initial literature search. Then, 165 duplicates were identified and excluded. Afterward, 404 studies were rejected based on relevance to the title abstract. After full-text screening, 10 articles were excluded, and six full-text articles and three abstract studies were finally included (Figure 1).

### 3.2 Characteristics of reviewed studies

Table 1 provides an overview of the study’s characteristics. The nine studies consist of 391 FD patients and 132 non-FD controls. Out of all the studies included, only one had a sample size greater than 100, with 257 cases (Tziatzios et al., 2021). The remaining studies had sample sizes below 100. Five studies were conducted in Australia (Zhong et al., 2016, 2017; Schooth et al., 2021, 2022; Shanahan et al., 2023), while others were conducted in Belgium (Wauters et al., 2021), China (Zheng et al., 2022), Greece (Tziatzios et al., 2021), and Japan (Fukui et al., 2020). This review contains one prospective cohort study (Wauters et al., 2021), one prospective cross-sectional study (Tziatzios et al., 2021), and seven case–control studies (Zhong et al., 2016, 2017; Fukui et al., 2020; Schooth et al., 2021, 2022; Zheng et al., 2022; Shanahan et al., 2023). The search did not include interventional studies carried out on FD patients. Out of all the studies included, three were explicitly presented as FD patients compared to healthy controls (Fukui et al., 2020; Wauters et al., 2021; Zheng et al., 2022), while the rest compared FD patients to non-FD patients (Zhong et al., 2016, 2017; Schooth et al., 2021, 2022; Tziatzios et al., 2021; Shanahan et al., 2023). Among the studies in which FD patients were compared with healthy controls, one study (Wauters et al., 2021) compared FD patients who had not been treated with PPI therapy (4-week healing dose) or other acid suppression < 3 months before inclusion (“FD-starters”) with FD patients who had persistent symptoms after >1 month of at least one daily dose of PPI (“FD-stoppers”) and healthy controls (Table 1).

**Table 1:** Characteristics of each study included.

Author, Year	Country	Study design	FD patients						Control group					
			Inclusion criteria	Exclusion criteria	*N* (*n*=)	Sex (M/F)	AGE (years): Mean ± SD	BMI (kg/m^2^): mean ± SD	Inclusion criteria	Exclusion criteria	*N* (*n*=)	Sex (M/F)	AGE (years): Mean ± SD	BMI (kg/m^2^): mean ± SD
Shanahan et al. (2023)	Australia	Case–control study (FD and Controls)	FD patients based on Rome IV or FD with additional irritable bowel syndrome-like symptoms were included. Only those who did not show any evidence of gastric/duodenal mucosal abnormalities, lesions, or structural changes (based on endoscopic and clinical histology findings) were included in the study.	Patients who reported the onset of GI symptoms after an acute infection have not been included in the study.	56	27/29	47 (17–77)^a^	25.2 ± 5.6	Control subjects were symptom-free (non-FD), with either documented iron deficiency (ID) with and without anemia or individuals undergoing screening following a positive fecal occult blood test. Only those who did not show any evidence of gastric/duodenal mucosal abnormalities, lesions, or structural changes (based on endoscopic and clinical histology findings) were included in the study.	Patients who reported the onset of GI symptoms after an acute infection have not been included in the study.	30	15/15	59 (22–74)^a^	27.5 ± 6.1
Zheng et al. (2022)	China	Case–control study (FD and Healthy Controls)	All enrolled patients were consistent with the diagnostic criteria of Rome IV.	Patients with *helicobacter pylori* infection were excluded.	20	3/17	36.6 ± 14.00	20.81 ± 12.98	The health control group excluded digestive systems and other systemic diseases by the examination of gastroscopy and abdominal ultrasound.	NR	5	1/4	28.8 ± 9.00	20.94 ± 13.10
Wauters et al. (2021)	Belgium	Prospective cohort study (FD-starters and FD-stoppers and Healthy Controls)	Patients with predominant FD symptoms were diagnosed according to Rome IV criteria.	All subjects had no *Helicobacter pylori*, active psychiatric, atopic, inflammatory, or metabolic conditions. Use of immunosuppressants, anti- or probiotics <3 months were exclusionary.	“FD-starters”: 28	“FD-starters”: 4/24	“FD-starters”: 27 (23.5–34.5)^b^	“FD-starters”: 22 (19-24)^b^ “	Age- and gender-matched healthy volunteers without GI symptoms were recruited as controls by advertisement.	All subjects had no *Helicobacter pylori*, active psychiatric, atopic, inflammatory, or metabolic conditions. Use of immunosuppressants, anti- or probiotics <3 months were exclusionary.	30	9/21	27 (24–33.5)^b^	23 (20–25.3)^b^
			“FD-starters”: no standard course of PPI therapy (4 weeks healing dose) and/or acid suppression <3 months before inclusion.		“FD-stoppers”: 19	“FD-stoppers”: 5/14	“FD-stoppers”: 32 (26.8–49.5)^b^	FD-stoppers”: 21.5 (20.8–24.3)^b^						
			“FD-stoppers”: refractory symptoms after >1 month of at least one daily dose of PPI.											
Zhong et al. (2017)	Australia	Case–control study (FD and Controls)	Patients presenting with FD based on Rome III.	NR	9	Two groups were matched for age, sex, and body mass index.			NR	Controls were screened for iron-deficiency anemia, and people with mucosal/ coeliac disease were excluded.	9	Two groups were matched for age, sex, and body mass index.		
Fukui et al. (2020)	Japan	Case–control study (FD and Healthy Controls)	The groups were age- and sex-matched. All 18 persons fulfilled the following criteria: no antibiotics, corticosteroids, immunosuppressants, and acid-suppressing agents were taken within 1 month. No mucosal inflammation or carcinoma was detected.	NR	11	6/5	56 (29–75)^a^	NR	The groups were age-and sex-matched. All 18 persons fulfilled the following criteria: no antibiotics, corticosteroids, immunosuppressants, and acid-suppressing agents were taken within 1 month. No mucosal inflammation or carcinoma was detected.	NR	7	4/3	52 (33–68)^a^	NR
Tziatzios et al. (2020)	Greece	Prospective cross-sectional study (FD and Controls)	Enrolled patients met Rome IV criteria for FD and reported no other miscellaneous gastrointestinal symptoms that could be indicative of potential overlap with other functional gastrointestinal disorders, i.e., IBS.	Patients aged <18 years, use of antibiotics or probiotics within the last 3 months, inability to provide informed consent, comorbidities, systemic diseases, diabetes mellitus, chronic constipation. Previous gastrointestinal surgeries with postsurgical structural changes, cirrhosis of any cause, and chronic pancreatitis or malignancies were excluded. Patients reporting other miscellaneous gastrointestinal complaints, i.e., diarrhea, malnutrition, weight loss indicative of SIBO, and current or recent use of PPI or H2 blockers, laxatives, and drugs that affect intestinal motility, were also excluded.	227	89/138	51.7 ± 13.8	NR	Individuals with a negative medical history of comorbidities undergoing upper GI endoscopy were considered eligible for inclusion. Those having neither pathological findings on endoscopy nor histological evidence of *Helicobacter pylori* (*H. pylori*) infection – identified as non-erosive reflux disease patients – were enrolled.	Individuals with any drug intake, current or recent (within the last 3 months), use of PPI or H2 blockers, and evidence of overlapping FD-type symptoms were excluded.	30	12/18	50.9 ± 14.6	NR
Zhong et al. (2016)	Australia	Case–control study (FD patients and Controls with no evidence of mucosal disease)	FD patients undergoing upper GI endoscopy were recruited after informed consent.	NR	9	NR	NR	NR	Individuals with no evidence of mucosal disease undergoing upper GI endoscopy	NR	9	NR	NR	NR
Schooth et al. (2022)	Australia	Case–control study (FD and non-FD controls)	NR	NR	6	NR	NR	NR	NR	NR	6	NR	NR	NR
Schooth et al. (2021)	Australia	Case–control study (FD and non-FD controls)	NR	NR	6	NR	NR	NR	NR	NR	6	NR	NR	NR

N, number; SD, Standard Deviation; M, male; F, female; NR, not reported; ^a^Data was presented as average age [minimum age, maximum age]; ^b^Data shown in median (interquartile range).

### 3.3 Microbiome assessment methods

Of the nine studies included in this systematic review, four studies (Zhong et al., 2016; Schooth et al., 2021, 2022; Shanahan et al., 2023) collected samples through duodenal biopsy only, and one (Fukui et al., 2020) performed upper gut brushing, while two others (Wauters et al., 2021; Zheng et al., 2022) used both duodenal biopsy and brushing. One study (Wauters et al., 2021) collected gastric and duodenal fluids, and another (Tziatzios et al., 2021) used duodenal fluid aspiration along with gastric biopsy. In terms of microbial assessment, 16S rRNA gene sequencing was used by most studies (*n* = 8) (Zhong et al., 2016, 2017; Fukui et al., 2020; Schooth et al., 2021, 2022; Wauters et al., 2021; Zheng et al., 2022; Shanahan et al., 2023). There were differences in the variable region sequenced among the eight studies that used bacterial 16S rRNA gene sequencing. One study (Wauters et al., 2021) used the V4 hypervariable region, one study (Fukui et al., 2020) used the V3-V4 hypervariable region, and two studies sequenced V6–V8 (Schooth et al., 2022; Shanahan et al., 2023), which was the commonly studied variable region (Table 2).

**Table 2:** Characteristics of gut microbiota assessments and factors influencing the microbiota.

Study	Type of specimen	Sample collection and storage	Microbiome assessment method	Alpha-diversity index					Beta-diversity index			Factors influencing microbiota
				Shannon	Simpson	Chao1	ACE	Observed species	Bray-Curtis distance	PCoA analysis	Amova analysis	
Shanahan et al. (2023)	Duodenal biopsies	Duodenal biopsies were taken from the second part of the duodenum utilizing the Brisbane Aseptic Biopsy device. Biopsy samples were immediately placed under aseptic conditions into a sterile tube containing RNA later (Qiagen). Samples were allowed to incubate at room temperature for 30 min, then frozen and stored at-80°C.	16S rRNA gene V6-V8 regions	NS	NR	↑	NR	NR	NS	NR	NR	PPI use: There were no differences between the two subgroups in the combined analysis.
												FD symptom burden: The relative abundances of predominant members of the Firmicutes, Bacteroidota, and Fusobacteriota phyla were linked to symptom burden in FD.
												Gastric emptying time: The study found inverse relationships between the relative abundances of Streptococcus and Prevotella and the relative abundance of Veillonella spp. with gastric emptying time.
												Habitual diet: The duodenal MAM is not significantly impacted by habitual diet.
Zheng et al. (2022)	Duodenal brushings and biopsies	The inclusive FD patients and healthy groups underwent the endoscopic examination, using a cell brush to brush the intestinal contents in the duodenum and using the biopsy to randomly clamp the mucosal tissue of the descending duodenum. The samples were stored in a − 80°C refrigerator.	16S rDNA gene sequencing	↑*	NR	NR	↑*	↑*	NR	Slight difference	Significant difference	NR
Wauters et al. (2021)	Duodenal brushings and biopsies	During upper GI endoscopy, aseptic biopsies from the second portion of the duodenum were collected using the sheathed and sealed Brisbane aseptic biopsy device (BABD), with additional precautions to avoid contamination. Next, a sterile brush was advanced while leaving the sheathed BABD in place for luminal brushing on the opposite side from where the biopsy sample was taken. Routine duodenal biopsies (for histology) and fluids (pH and bile salts) were collected in all subjects at baseline and follow-up. Samples were immediately snap-frozen and stored at −80°C.	16S rRNA gene V4 hypervariable region	Brush: ↓	Brush: ↓	Brush: ↓	NR	Brush: ↓	NR	NR	NR	PPI use: Baseline differences and effects of short-term PPI therapy were only found for specific luminal genera and diversity, while long-term PPI therapy may have a certain effect on duodenal Dysbiosis, with stable duodenal luminal and mucosal bacterial communities in the absence of PPI therapy.
				Biopsy: ↓	Biopsy: ↓	Biopsy: ↑		Biopsy: ↑				
Zhong et al. (2017)	NR	NR	16S rRNA gene sequencing and qPCR	NR	NR	NR	NR	NR	NR	NR	NR	Quality of life: There is a negative correlation between duodenal mucosal bacterial load and reported quality of life.
												Symptom responses: More severe symptom responses to the standardized meal positively correlated with mucosal bacterial load.
Fukui et al. (2020)	Upper gut brushings: the middle of the esophagus, gastric body, gastric antrum, and descending portion of the duodenum	The upper gut samples were collected from the middle of the esophagus, gastric body, gastric antrum, and descending portion of the duodenum by upper endoscopy. Under endoscopic examination, the samples were taken by gently brushing each 10 times with a Cytology brush. Each sample was placed into 500 μL of sterile phosphate buffer saline. The samples were frozen and stored at −80°C in a freezer.	16S rRNA gene V3–V4 hypervariable region	NS	NR	NS	NR	NS	NR	Significant difference	NR	Gastrointestinal symptoms: In the species level in Streptococcus, the relative abundance of OTU 90 (the most abundant sequence of *Streptococcus infantis*) was positively correlated with PDS and EPS scores. OTU 90 is a bacterium strongly correlated with upper gastrointestinal symptoms.
Tziatzios et al. (2020)	Duodenal juice aspirated and gastric biopsy	From the greater and lesser curvature of the antrum, incisura, and corpus, researchers extracted five gastric biopsy specimens, which were placed into separate vials and grouped by site. The endoscopist passed the ERCP catheter through the biopsy channel of the endoscope and advanced it into the third and fourth portions of the duodenum for collection of the small bowel aspirate. Small bowel aspirates were quantitatively cultured under aerobic conditions. Plates were cultured for 24 h at 37°C under aerobic conditions.	NR	NR	NR	NR	NR	NR	NR	NR	NR	FD subtypes: Among dyspeptics, SIBO is more frequent in PDS and overlaps PDS-EPS subgroups, but not EPS alone.
Zhong et al. (2016)	Duodenum biopsies	NR	16S rRNA gene sequencing and qPCR	NR	NR	NR	NR	NR	NR	NR	NR	Symptom responses: When the duodenal mucosal bacterial load was assessed, an increase in bacterial levels was found to be associated with augmented meal-related symptom responses during the nutrient challenge.
												Disease-specific quality of life scores: Increased bacterial load was also negatively correlated with disease-specific quality of life scores.
Schooth et al. (2022)	Duodenum biopsies	Duodenal biopsy samples collected with the Brisbane Aseptic Biopsy Device were placed in an anaerobically prepared cryopreservative solution and stored at −80°C. The tissue was later aseptically transferred into a broth medium simulating the duodenal habitat (DHSM-1), cultured for 24 h, and then subsampled for storage as described above. These outgrowth cultures of the duodenal MAM were then used to inoculate 10-mL volumes of DHSM-1 broth for growth studies.	16S rRNA gene V6-V8 region	NR	NR	NR	NR	NR	NR	NR	NR	NR
Schooth et al. (2021)	Duodenum biopsies	Biopsies were collected from FD and non-FD control subjects using the Brisbane aseptic biopsy device and stored anaerobically. Biopsies were transferred to a habitat-simulating medium, incubated at 37°C, and microbial growth monitored. These cultures were sampled and stored anaerobically. Stocks were then used on two occasions to inoculate fresh media and cultured as described above.	16S rRNA gene sequencing	NR	NR	NR	NR	NR	NR	NR	NR	NR

↑, higher relative to comparison group; ↓, lower relative to comparison group; *, significant difference; NS, nonsignificant difference; NR, not reported.

### 3.4 Alterations in the duodenal microbiota composition of FD patients

Three studies (Fukui et al., 2020; Zheng et al., 2022; Shanahan et al., 2023) have delineated variations in the duodenal microbiota at the phylum level. Two studies (Zheng et al., 2022; Shanahan et al., 2023) found an increase in the amounts of *Fusobacteria* in FD patients, whereas a third study (Fukui et al., 2020) showed no difference compared to healthy controls. Findings from one study (Zheng et al., 2022) suggest that the phylum *Firmicutes* constitutes a substantial proportion of the total sequences in both FD patients and healthy individuals and represents the dominant bacteria of the duodenal flora. One study (Fukui et al., 2020) demonstrated a significant increase in the phylum *Firmicutes* in FD patients. While another study (Shanahan et al., 2023) did not observe a marked difference in the relative abundance of *Firmicutes* between FD patients and healthy controls, it did discern a correlation between the abundance of this phylum and the severity of FD symptoms, with an escalation in *Firmicutes* accompanying an increased symptom burden in FD patients. In one study (Zheng et al., 2022), the phylum *Bacteroidetes* and *Proteobacteria* were identified as the dominant species of duodenal flora. However, three investigations (Fukui et al., 2020; Zheng et al., 2022; Shanahan et al., 2023) collectively found no significant differences in the abundance of *Bacteroidetes* and *Proteobacteria* between FD patients and the control group. Additionally, one study (Zheng et al., 2022) remarked that there was no significant difference in the composition of duodenal flora at the phylum level between FD patients and healthy controls.

At the genus level, there were three Australian studies (Zhong et al., 2016, 2017; Shanahan et al., 2023) demonstrating a significantly decreased number of bacteria in the genus *Actinomyces*. The genus *Selenomonas* was evaluated in three studies (Wauters et al., 2021; Zheng et al., 2022; Shanahan et al., 2023). Of these, one study (Zheng et al., 2022) showed a significant decrease in the amount of the genus *Selenomonas* in FD patients, while the other two studies (Wauters et al., 2021; Shanahan et al., 2023) showed a non-significant trend toward decreased amounts. The results of a study in China (Zheng et al., 2022) showed significantly increased numbers of the genera *Staphylococcus* and *Peptostreptococcus* in FD patients. In contrast, an Australian study (Shanahan et al., 2023) showed a non-significant trend of increase for these genera. Another study identified the genus *Staphylococcus* as a predominant component of the duodenal microbiota in FD patients (Zhong et al., 2016). Two studies evaluated the genus *Alloprevotella* in 76 FD patients (35 healthy controls) and showed a significant increase in one study (Zheng et al., 2022) and a non-significant increase in another (Shanahan et al., 2023). Genus *Haemophilus* showed a decreasing trend in two studies (Wauters et al., 2021; Shanahan et al., 2023), whereas in one study of 9 FD patients compared to 9 healthy controls (Zhong et al., 2017), the relative abundance of the genus *Haemophilus* was not found to be significantly different. Another study from China (Zheng et al., 2022) found that the genus *Haemophilus* was one of the most important microorganisms in the duodenal flora of both FD patients and healthy controls. Furthermore, two studies (Zhong et al., 2017; Shanahan et al., 2023) compared a total of 65 FD patients with 39 healthy controls and concluded that the microbial counts of genus *Megasphaera* showed a decreasing trend in FD patients.

At the genus level, there were conflicting results of cumulative evidence for several significant microbiota. Three studies (Zhong et al., 2017; Zheng et al., 2022; Shanahan et al., 2023) have identified the genus *Streptococcus* as the dominant organism in the duodenal flora of both FD patients and healthy controls. A Japanese study (Fukui et al., 2020) showed a significant increase in the duodenal genus *Streptococcus* counts in FD patients compared to controls, and the results of another study (Zhong et al., 2017) also suggested a non-significant trend toward an increase, while the result of an Australian study (Shanahan et al., 2023) showed a non-significant decreasing trend of this genus. Genus *Prevotella* was also identified as a dominant species within the duodenal microbiota in three studies (Zhong et al., 2016; Zheng et al., 2022; Shanahan et al., 2023). However, a significant decrease in the genus *Prevotella* was found in one study (Zhong et al., 2017), and a non-significant increase was found in another study (Shanahan et al., 2023). A study conducted in China (Zheng et al., 2022) found that the genus *Fusobacterium*, genus *Neisseria*, and genus *Porphyromonas* were significant microbiota in the duodenum of both healthy individuals and FD patients at the genus level. Another study (Zhong et al., 2016) also identified the genus *Porphyromonas* as an important component of the duodenal flora in FD patients. Both the genera *Neisseria* and *Porphyromonas* had the same trend of alteration in three articles (Zhong et al., 2017; Wauters et al., 2021; Shanahan et al., 2023). In two of the articles (Zhong et al., 2017; Wauters et al., 2021), the relative abundance of these two genera in FD patients showed a decreasing trend, whereas in another article (Shanahan et al., 2023), their relative abundance exhibited an increasing trend. Four studies (Zhong et al., 2017; Schooth et al., 2021; Wauters et al., 2021; Shanahan et al., 2023) assessed the genus *Fusobacterium*. One study found a significantly increased amount of this genus in FD patients (Shanahan et al., 2023), while two studies (Schooth et al., 2021; Wauters et al., 2021)showed a trend for a decreased amount, and the other (Zhong et al., 2017) revealed a non-significant difference.

**Figure 2:**
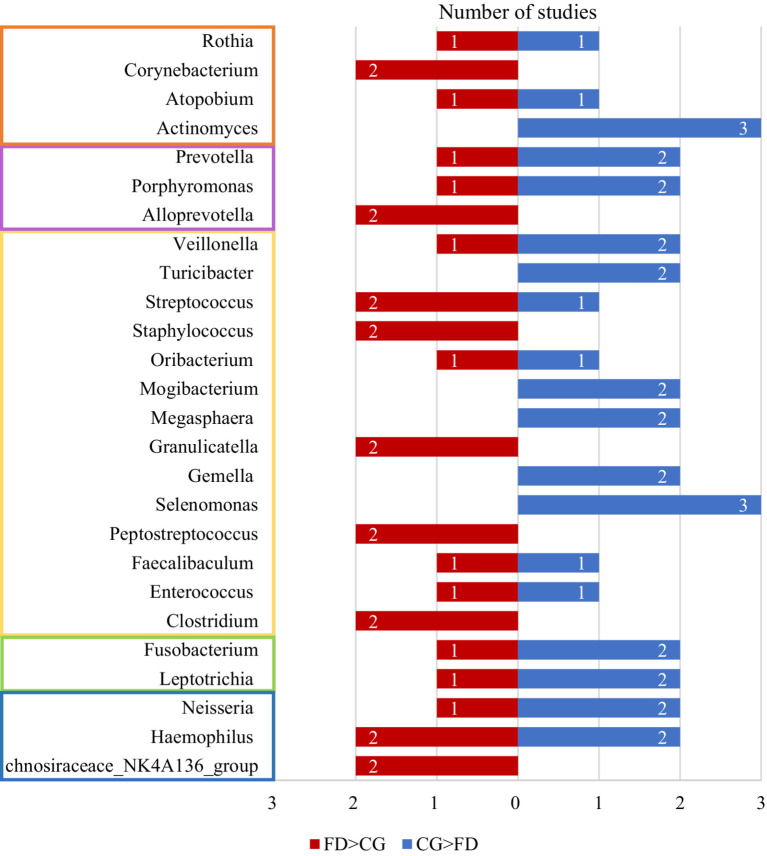
The number of studies reporting differences in bacterial genera between the FD group and the CG group across two or more studies. The blue bars represent the number of studies where the abundance of bacterial genera was higher in the CG group compared to the FD group, while the red bars indicate the number of studies where the abundance was higher in the FD group than in the CG group. Genera within the orange frame belong to the *Actinobacteria*, those in the purple frame to the *Bacteroidetes*, those in the yellow frame to the *Firmicutes*, those in the green frame to the *Fusobacteria*, and those in the blue frame to the *Proteobacteria*. CG, control group; FD, functional dyspepsia.

Summarizing the abundance changes of genera reported in two or more studies and categorizing them at the phylum level, it can be observed that genera under the phylum *Firmicutes* exhibit the highest number of changes between FD and non-FD control groups, followed by those under the phylum *Actinobacteria* (Figure 2; Table 3).

**Table 3:** The taxonomic changes to duodenal microbiota.

**Study**	**Trend**	**Changes at the taxonomic level**					
		**Phylum level**	**Class level**	**Order level**	**Family level**	**Genus level**	**Species level**
Shanahan et al. (2023)	↑	TM7 Proteobacteria Fusobacteria* Bacteroidetes	NR	NR	NR	Fusobacterium*, *Alloprevotella*, *Acidovorax*, *Prevotella*, *Ralstonia*, *TM7x*, *Porphyromonas*, *Cloacibacterium*, *Diaphorobacter*, *Granulicatella*, *Neisseria*, *Staphylococcus*, *Parvimonas*, *Comamonas*, *Leptotrichia*, *Atopobium*, *Acinetobacter*, *Peptostreptococcus*, *Oribacterium*, *Methylophilus*, *Actinobacillus*	NR
	↓	*Firmicutes* *Actinobacteria*	NR	NR	NR	*Mogibacterium*, *Faecalibaculum*, *Actinomyces*, *Veillonella*, *Enterococcus*, *Solobacterium*, *Coriobacteriaceae*_UCG-002, *Haemophilus, *Dubosiella*, *Stomatobaculum*, *Gemella*, *Megasphaera*, *Delftia*, *Allobaculum*, *Lactobacillus*, *Stenotrophomonas*, *Turicibacter*, *Rothia*, *Lachnospiraceae*_NK4A136_group, *Selenomonas*, *Streptococcus*, *Bulleidia*, Helicobacter*	NR
	Dominant bacteria	NR	NR	NR	NR	NR	NR
Zheng et al. (2022)	↑	*Fusobacteria*	NR	NR	NR	Alloprevotella*, *chnosiraceace*_NK4A136_group*, Corynebacterium*, [*Eubacterium*]_nodatum_group*, Faecalibaculum*, Lachnoclostridium*, Lautropia*, Peptostreptococcus*, Staphylococcus*, Sutterella* LEfSe analysi: o_Corynebacteriales*, c_Clostridia*, o_*Clostridia*_vadinBB60_group*, o_Sphingomonadales*, c_Alphaproteobacteria*, o_Oscillospirales*, f_Sphingomonadaceae*, s_*Prevotella*_intermedia*, s_*Prevotella*_jejuni*, f_Ruminococcaceae*, f_Peptostreptococcaceae*, g_Peptostreptococcus*, s_*Haemophilus*_influenzae*	NR
	↓	NR	NR	NR	NR	Catonella* LEfSe analysi: g_Johnsonella*, g_Catonella*, g_Selenomonas*	NR
	Dominant bacteria	*Firmicutes Bacteroidetes* *Proteobacteria*	NR	NR	NR	*Streptococcus*, *Actinobacillus*, *Neisseria*, *Fusobacterium*, *Prevotella*, *Haemophilus*, *Porphyromonas*	NR
Wauters et al. (2021)	↑	NR	NR	NR	NR	NR	NR
	↓	NR	NR	NR	NR	Fusobacterium, *Haemophilus*, Neisseria*, Porphyromonas*, Selenomonas	NR
	Dominant bacteria	NR	NR	NR	NR	NR	NR
Zhong et al. (2017)	↑	NR	NR	NR	Paraprevotellaceae	*Streptococcus*, *Carnobacterium*, *Corynebacterium*, *Gemella*	NR
	↓	NR	NR	NR	NR	Actinomyces*, Atopobium*, Leptotrichia*, Prevotella*, Veillonella*, *Megasphaera*, *Neisseria*, *Porphyromonas*	NR
	Dominant bacteria	NR	NR	NR	NR	*Streptococcus*	NR
Fukui et al. (2020)	↑	Firmicutes* Actinobacteria	NR	NR	NR	Streptococcus*, *Veillonella*	NR
	↓	*Bacteroidetes* *Fusobacteria*	NR	NR	NR	*Oribacterium*, *Lactobacillus*, *Granulicatella*	NR
	Dominant bacteria	*Proteobacteri* *Bacteroidetes* *Firmicutes* *Fusobacteria* *Actinobacteria*	NR	NR	NR	NR	NR
Tziatzios et al. (2020)	↑	NR	NR	NR	NR	NR	*Escherichia coli, Klebsiella pneumonia, Acinetobacter baumannii, Serratia marscecens, Klebsiella spp, Pseudomonas* *aeruginosa, Staphylococcus aureus, Enterobacter cloacae*
	↓	NR	NR	NR	NR	NR	NR
	Dominant bacteria	NR	NR	NR	NR	NR	NR
Zhong et al. (2016)	↑	NR	NR	NR	NR	NR	NR
	↓	NR	NR	NR	NR	Actinomyces*.	NR
	Dominant bacteria	NR	NR	NR	NR	FD Group: *Prevotella*, *Streptococcus*, *Porphyromonas*	NR
Schooth et al. (2022)	↑	NR	NR	NR	NR	NR	*V. atypica*, *V. rogosae*
	↓	NR	NR	NR	NR	NR	*Veillonella ratti/cricetid, Veillonella parvula*
	Dominant bacteria	NR	NR	NR	NR	NR	NR
Schooth et al. (2021)	↑	NR	NR	NR	NR	*Enterococcus*, *Granulicatella*	NR
	↓	NR	NR	NR	NR	*Dialister*, *Fusobacterium*, *Gemella*, *Leptotrichia*, *Leuconostoc*, *Mogibacterium*	NR
	Dominant bacteria	NR	NR	NR	NR		NR

Legend:↑, higher relative to comparison group; ↓, = lower relative to comparison group; *, significant difference; NR, not reported.

### 3.5 Duodenal microbiota diversity in FD patients

Four articles (Fukui et al., 2020; Wauters et al., 2021; Zheng et al., 2022; Shanahan et al., 2023) compared the alpha diversity of FD patients and controls by using different indices and methods. Four studies (Fukui et al., 2020; Wauters et al., 2021; Zheng et al., 2022; Shanahan et al., 2023) reported the Shannon index, three reported the Chao1 index (Fukui et al., 2020; Wauters et al., 2021; Shanahan et al., 2023), three reported observed species (Fukui et al., 2020; Wauters et al., 2021; Zheng et al., 2022), one reported the ACE index (Zheng et al., 2022), and one reported the Simpson index (Wauters et al., 2021). Only one study (Zheng et al., 2022) showed a significant increase in alpha diversity in FD patients, while three other studies (Fukui et al., 2020; Wauters et al., 2021; Shanahan et al., 2023) found no significant difference in alpha diversity between FD patients and the controls.

Three studies analyzed the β diversity of duodenal microbiota in FD patients and their control groups (Fukui et al., 2020; Zheng et al., 2022; Shanahan et al., 2023). Two studies showed the result through the β diversity principal coordinates analysis (PCoA) (Fukui et al., 2020; Zheng et al., 2022). One of the studies (Fukui et al., 2020) showed that there were microbial structural differences between FD and healthy subjects when PCoA was used. The other study (Zheng et al., 2022) based on the results of PCoA showed that the duodenal flora of FD patients and healthy people did not show a clear separate trend, but further Amova analysis of the research revealed that there was a significant difference between the two groups, indicating that the structure of duodenal flora changed in FD patients. One study (Shanahan et al., 2023) independently demonstrated an insignificant difference in β diversity (Bray-Curtis distance; Table 2).

### 3.6 Factors associated with duodenal microbiota alterations

Two studies have described the relationship between changes in the duodenal microbiota and the use of PPI in FD patients (Wauters et al., 2021; Shanahan et al., 2023). One of the studies showed that (Shanahan et al., 2023) the impacts of PPI use on the duodenal mucosa-associated microbiota (MAM) can be variable but overall limited. The other study (Wauters et al., 2021) showed that baseline differences and effects of short-term PPI therapy were only found for specific luminal genera and diversity, while long-term PPI therapy may have a certain effect on duodenal dysbiosis. Four studies (Zhong et al., 2016, 2017; Fukui et al., 2020; Shanahan et al., 2023) investigated the correlation between duodenal flora changes and FD symptoms burden. A study (Shanahan et al., 2023) suggested that the relative abundances of predominant members of the phylum *Firmicutes* and *Bacteroidota* were linked to symptom burden in FD, in which the relative abundance of taxa affiliated with *Firmicutes* increased with FD symptom burden, whereas taxa affiliated with *Bacteroidota* decreased. The other two studies (Zhong et al., 2016, 2017) suggested that more severe symptom responses to the standardized meal positively correlated with mucosal bacterial load. These two studies (Zhong et al., 2016, 2017)also explored the relationship between duodenal microbiota dysbiosis and the quality of life in FD patients and found a negative correlation exists between duodenal mucosal bacterial load and reported quality of life (Table 2).

### 3.7 Quality of the evidence

The quality of evidence was assessed by the Newcastle-Ottawa Scale for the six full-text articles, with two of the six articles scoring 7, one scoring 6, and three scoring 5, giving an overall moderately high level of article quality. Among the six full-text articles reviewed, all studies had clear definitions of the study and control populations. Regarding the study population, four studies (Tziatzios et al., 2021; Wauters et al., 2021; Zheng et al., 2022; Shanahan et al., 2023) explicitly diagnosed and included FD patients based on the Rome IV criteria; one study used the Rome III criteria; and only one study did not specify the exact diagnostic criteria for FD patients but established standards for endoscopic findings in FD patients. Additionally, five studies (Fukui et al., 2020; Tziatzios et al., 2021; Wauters et al., 2021; Zheng et al., 2022; Shanahan et al., 2023) mentioned that both FD and control groups underwent endoscopic examination to rule out gastric/duodenal mucosal abnormalities, lesions, or structural changes, with only one study (Zhong et al., 2017) not explicitly addressing this point. For the control groups, three studies (Fukui et al., 2020; Wauters et al., 2021; Zheng et al., 2022) used healthy controls, and the other three (Zhong et al., 2017; Tziatzios et al., 2021; Shanahan et al., 2023) used non-FD controls who did not exhibit FD symptoms and were confirmed to have no upper gastrointestinal mucosal lesions via endoscopy. All studies assessed whether baseline data, mainly age and gender, were statistically different between the two groups, with only one study (Shanahan et al., 2023) having a significantly younger group of FD patients than the control group. None of the trials reported whether blinding was used (Table 4).

**Table 4:** Quality of the included studies by the NOS.

Study	Selection				Comparability		Exposure			Score
	Is the case definition adequate?	Representativeness of the cases	Selection of controls	Definition of controls	Comparability of baseline characteristic 1 (age)	Comparability of baseline characteristic 2 (sex)	Ascertainment of exposure	The same method of ascertainment for cases and controls	Non-response rate	
Shanahan et al. (2023)	*	*	*	*	*	*	NR	NR	*	7*
Zheng et al. (2022)	*	*	NR	*	*	*	NR	NR	*	6*
Wauters et al. (2021)	*	*	*	*	*	*	NR	NR	*	7*
Zhong et al. (2017)	*	NR	NR	*	*	*	NR	NR	*	5*
Fukui et al. (2020)	*	*	*	*	*	*	NR	NR	*	7*
Tziatzios et al. (2020)	*	*	*	*	*	*	NR	NR	*	5*

*, indicates the quality of the studies; NR, not reported.

## 4 Discussion

The duodenum is currently postulated to play an important role in the pathogenesis of FD. The increased permeability and microinflammation of the duodenal mucosa implicated in the etiology of FD are thought to be associated with alterations in the microbial community (Miwa et al., 2019). FD has been recognized as a disorder of brain-gut interaction, implicating the duodenal microbiota as a potentially significant yet understudied player in the brain-gut dialog. We present the first comprehensive review of existing studies on the duodenal microbiota in FD, attempting to elucidate the composition and characteristics of the duodenal microbiota in FD patients and establish a correspondence between symptom production and disease pathogenesis. The results of this study showed that the diversity of the duodenal microbiota in FD patients was not significantly different from that of non-FD controls but tended to increase and that changes in the duodenal microbiota of FD patients correlated with patient symptom burden and affected patient quality of life. In FD patients, a number of duodenal microbiota have a relatively significant upward trend, and we consider this type of microorganism to be potentially harmful, including the phylum *Fusobacteria*, the genera *Alloprevotella*, *Corynebacterium*, *Peptostreptococcus*, *Staphylococcus*, *Clostridium*, *Streptococcus,* and others. Flora with a more pronounced downward trend in FD patients include the genera *Actinomyces*, *Gemella*, *Haemophilus*, *Megasphaera*, *Mogibacterium*, *Selenomonas*, and others, and we believe that there may be potentially beneficial bacteria for FD patients in these microbiotas. Furthermore, alterations in specific microbial taxonomic groups and their implications for the brain-gut axis interactions in patients with functional dyspepsia warrant attention.

### 4.1 Correlation of *Streptococcus* and *Prevotella* abundance with FD symptoms

The results of a Japanese study (Fukui et al., 2020) observed a significant increase in the abundance of *Firmicutes* in FD compared to healthy controls in all sites of the upper gut, and the evaluation of taxonomic changes of each genus in the *Firmicutes* revealed that only genus *Streptococcus* was significantly increased in all sites in the upper gut in FD compared to healthy controls, and this study also found a positive correlation between the relative abundance of genus *Streptococcus* and upper gastrointestinal symptoms in FD patients. This finding is consistent with another included Australian study (Shanahan et al., 2023), which also found that the relative abundance of the *Firmicutes* was significantly higher in FD patients and the relative abundance of taxa affiliated with the *Firmicutes* increased with FD symptom burden, and this positive correlation trend continued when the dominant genus of this phylum (i.e., *Streptococcus*) was examined. This suggests that *Streptococcus* spp. may be the main suspected genus closely associated with symptoms in FD patients. In contrast, the results of the study also showed that the relative abundance of *Prevotella* spp. was negatively correlated with the severity of FD symptom burden. In addition, both experiments (Zhong et al., 2017; Shanahan et al., 2023) indicated that the relative abundance of genus *Streptococcus* and genus *Prevotella* in FD patients was inversely related.

The genera *Streptococcus* and *Prevotella* are the main bacteria of the upper gastrointestinal tract. Interestingly, one study found that the changes in the relative abundance of the genera *Streptococcus* and *Prevotella* are thought to correlate significantly with changes in duodenal pH, and the two genera show an opposite trend in correlation with duodenal pH, with *Streptococcus* predominantly exhibiting a positive correlation and *Prevotella* demonstrating a negative association with the changes in duodenal pH (Seekatz et al., 2019). This seems to confirm that the genera *Streptococcus* and *Prevotella* are inversely characterized in the duodenum of FD patients and that changes in the relative abundance of the two genera correlate with the changes in duodenal pH in FD patients. There is a strong correlation and an interactive relationship between the duodenal microbiota and the pH of the duodenal contents. Large fluctuations in environmental pH may select genera such as *Streptococcus*, which can also regulate intracellular pH (Abuhelwa et al., 2017; Seekatz et al., 2019).

The growth of microorganisms, including the genera *Streptococcus* and *Prevotella*, can lead to pH changes by mediating the metabolism of short-chain fatty acids (SCFA; Zoetendal et al., 2012). *Streptococcus* has been demonstrated to elevate concentrations of lactate and butyrate within the duodenum, consequently lowering the pH of the duodenal environment (Zoetendal et al., 2012). *In vitro* studies (Chen et al., 2017) have corroborated that *Prevotella* exhibits a heightened capacity for fiber utilization and serves as an efficient producer of propionate from arabinoxylan and oligofructose. The enhanced capacity for fiber degradation makes the genus *Prevotella* potentially beneficial for glucose homeostasis and host metabolism (Tett et al., 2021). Furthermore, *Prevotella* has been shown to augment bile acid metabolism (Péan et al., 2020), thereby exerting influence on the duodenal pH. Alterations in duodenal pH are posited to contribute to the pathogenesis of FD, with FD patients demonstrating evidence of increased duodenal acid exposure (Bratten and Jones, 2009). Additionally, the sensitivity of the duodenal mucosa to acid is thought to be associated with FD, and acid exposure at the duodenal site in FD patients is believed to worsen FD symptoms (Bratten and Jones, 2009; Ishii et al., 2010).

Based on the included studies, we observed that the duodenal microbiota of FD patients exhibited negatively correlated changes in the genera *Streptococcus* and *Prevotella*. These variations in the two genera may be closely associated with the onset of FD symptoms. The change in duodenal pH may play a significant role in this process.

### 4.2 Staphylococcal enterotoxin may induce duodenal immune inflammation leading to functional dyspepsia

According to the results from the included studies, the genus *Staphylococcus* exhibits a notably increased trend in the duodenal microbiota of FD patients. Furthermore, one study (Tziatzios et al., 2021) indicated that the proportion of *Staphylococcus aureus* in small intestinal aspirates is higher in FD patients with small intestinal bacterial overgrowth (SIBO) compared to non-FD patients. Duodenal inflammation is considered a novel target in the pathogenesis of FD, and microinflammation in the form of local immune cell infiltration plays an important role in the pathogenesis of FD (du et al., 2018; Wauters et al., 2020b). The increase in the genus *Staphylococcus* presence observed in this study may provide a new avenue of investigation into the mechanisms underlying FD. Previous studies have demonstrated a correlation between the overgrowth of *Staphylococcus* species and inflammatory bowel disease (IBD), which is attributed to the inflammation induced by staphylococcal superantigens (Jun et al., 2003; Collado et al., 2008). *Staphylococcus aureus* is a member of the genus *Staphylococcus*, and the staphylococcal enterotoxin (SE) produced by *S. aureus* is considered to be the causative agent of human food poisoning as well as a potent immune superantigen that readily crosses the intact intestinal epithelium, inducing the proliferation of activated T cells and the release of large amounts of pro-inflammatory cytokines (White et al., 1989; Moretó and Pérez-Bosque, 2009; Principato and Qian, 2014). Staphylococcal enterotoxin A (SEA) produced by *Staphylococcus aureus* has been shown to elicit a rapid immunological response in the stomach and duodenum of rats, and the duodenum displayed a greater leukocytic response than the stomach to SEA, while studies have shown that SEA does not induce gastrointestinal mucous membrane damage encompassing edema, cytolysis, tissue sloughing, luminal necrotic tissue, or alternations in epithelial mitotic (Beery et al., 1984). Liu et al. (2022) reported that SEA significantly upregulated the expression of NLRP3 inflammasome-associated proteins and downregulated the expression of tight junction (TJ) proteins, which triggered the mitogen-activated protein kinase (MAPK) and nuclear factor kappa-B (NF-κB) signaling pathways in jejunal tissue, inducing intestinal barrier dysfunction and small bowel injury in mice. Staphylococcal enterotoxin B (SEB) was found to evoke significant increases in myeloperoxidase (MPO), macrophage infiltration, T-cell activation, and perturbed epithelialion transport and cause low-grade inflammation of the colonic lumen (Jun et al., 2003). Studies (Moretó and Pérez-Bosque, 2009; Pérez-Bosque and Miquel, 2010) have also shown that SEB exposure decreases the expression of mucosal tight-junction and adherent-junction proteins, leading to increased mucosal permeability and intestinal secretion.

In conclusion, although the definitive studies elucidating the mechanistic role of duodenal *Staphylococcus aureus* in FD are lacking, SE can damage the intestinal mucosal barrier, activate duodenal immunity, and induce an inflammatory response, and its potential inducing effect on the pathogenesis of FD may be worthy of attention.

### 4.3 *Selenomonas* as a potential “beneficial bacterium” in the duodenum of functional dyspepsia patients

Several studies have found (Ricke et al., 1996; Jordan and Kurt, 2010) that *Selenomonas* have multiple carbon flow routes for carbohydrate catabolism and ATP generation and that catalytically efficientβ-D-xylosidase from *Selenomonas* can hydrolyze cellulose, and *Selenomonas* is thought to promote nutrient digestibility. In addition, recent research has found that (Kim et al., 2022) treatment during the asthma sensitization period with *Selenomonas sputigena* resulted in a significant reduction in airway hyperresponsiveness and a decrease in inflammatory cells present in bronchoalveolar lavage fluid, suggesting that the *Selenomonas* may be able to mitigate the severity of asthmatic phenotypes. In terms of gastrointestinal diseases, the researchers found that the symptoms of the patients with functional constipation (FC) and comorbid depression and anxiety were improved after fecal microbiota transplantation. An increase in the abundance of *Selenomonas* within FC patients’ gut microbiota has been noted, leading to the hypothesis that changes in the prevalence of this bacterial genus may be involved in the pathogenesis of constipation presenting with psychiatric symptoms (Yang et al., 2023).

The above results suggest that *Selenomonas* may have a potential “protective” effect on the human body. Current research on *Selenomonas* predominantly focuses on ruminant animals, with a relative paucity of studies elucidating its mechanisms of action in humans. However, in the present study, the genus *Selenomonas* exhibited a relatively significant downward trend in the duodenal microbiota of FD patients. Although it is unclear whether this is the cause or the result of FD, considering the characteristics of this genus, *Selenomonas* may represent a potentially beneficial bacterial genus for FD patients.

### 4.4 The role of duodenal microbial dysbiosis in gut–brain interactions in functional dyspepsia

Functional GI disorders are classified by the Rome IV criteria as disorders of gut–brain interaction with contributions of both altered brain processing and luminal changes, including dysbiosis (Drossman and William, 2016). Emerging data increasingly points to the duodenum as a key integrator in the generation of dyspepsia symptoms. In patients with dyspepsia, irritation of the duodenum is thought to cause disturbances in duodenal-gastric feedback, leading to gastric motility dysfunction, which in turn causes dyspeptic symptoms (Walker and Nicholas, 2017; Miwa et al., 2019). A study from Leuven (Cirillo et al., 2015) demonstrated a correlation between duodenal eosinophils or mast cells and the Ca^+^ transient amplitude under high-K^+^ depolarization or electrical pulses, suggesting a role for duodenal inflammation in FD neuronal signal transmission.

Current evidence (Fukui et al., 2020) suggests that dysbiosis of the duodenal MAM is considered to play a pivotal role in the development of symptoms in FD patients. The results of a systematic review and meta-analysis (Gurusamy et al., 2021b) further corroborate a link between FD and small intestinal bacterial overgrowth. Additionally, relevant preclinical studies (de Palma et al., 2015) have indicated that stress can induce dysregulation of the gut microbiota, thereby impacting the function and behavior of the central nervous system. Thus, consideration must be given to the potential stimulation of the duodenal mucosa and its neuronal signal transmission by duodenal microbiota and their metabolic products, which may consequently contribute to the pathogenesis of FD through gastrointestinal dysmotility.

The composition and abundance fluctuations of the gut microbiota have been associated with compromised mucosal surface integrity (Zhou et al., 2022). In FD patients, the duodenal mucosa exhibits compromised mucosal integrity and increased permeability, which is believed to permit luminal triggers to initiate local and systemic immune cascades, leading to alterations in neuronal signal transduction and the consequent manifestation of dyspeptic symptoms (Nojkov et al., 2020; Wauters et al., 2020a). Enterotoxins released by *Staphylococcus* are recognized to disrupt small intestinal mucosal barrier function, invade the intestinal mucosa, and elicit immune and inflammatory responses. Genus *Alloprevotella* has also been identified as a key bacterium capable of inducing colonic mucosal damage (Wang et al., 2021). Studies by Zhao et al. (2021) have demonstrated that sodium caprylate (SC) treatment increased the populations of *Prevotella_9* in the ileum and *Lachnoclostridium* and *Roseburia* in the colon but decreased the abundances of *Streptococcus* and *Enterococcus* in the ileum and *Lactobacillus* and *Clostridium_sensu_stricto_1* in the colon, thereby ameliorating intestinal barrier function and maintaining gut health.

The gut microbiota exerts a pivotal influence on the nervous, neuroendocrine, and metabolic systems, producing microbial metabolites, signaling molecules, and hormones with potential implications for alterations in brain signaling (Cowan et al., 2020; Vanuytsel et al., 2023). Short-chain fatty acids (SCFAs) represent metabolites of gastrointestinal microbial communities and are often considered key mediators of communication between the central nervous system and the gut. SCFAs can induce the secretion of glucagon-like peptide 1 (GLP-1) and peptide YY (PYY), γ-aminobutyric acid (GABA), and other hormones capable of transmitting stimuli to the central nervous system via the circulatory system or the vagus nerve pathway (Silva et al., 2020). SCFAs are primarily produced by bacteria belonging to genera such as *Prevotella*, *Streptococcus*, *Lactobacillus*, and *Bifidobacterium*, utilizing dietary fibers, resistant starch, oligosaccharides, and other intestinal compounds as substrates (Markowiak-Kopeć and Katarzyna, 2020). Among the taxa exhibiting a declining trend in this study, genus *Prevotella* demonstrates robust SCFA production capability and high fiber utilization efficiency, serving as proficient propionate producers from arabinoxylan and fructooligosaccharides (Chen et al., 2017). Furthermore, studies (Zhao et al., 2018) have revealed positive correlations between the increased abundance of genera *Prevotella*, *Lactobacillus*, and *Alistipes* and concentrations of saturated long-chain fatty acids (SLCFAs) in the gut, where elevated SLCFA levels promote intestinal smooth muscle contraction, leading to increased gut motility; however, the precise mechanisms through which SLCFAs enhance gastrointestinal motility remain unclear. The gastrointestinal microbiota also modulates various facets of the gut–brain axis (GBA) through serotonin metabolism. Serotonin, derived solely from tryptophan, serves as a critical monoaminergic neurotransmitter involved in central nervous transmission and intestinal physiological functions (Gao et al., 2020). Numerous bacterial strains, including those from *Lactococcus*, *Lactobacillus*, *Streptococcus*, *Escherichia coli*, and *Klebsiella* genera, have been documented to possess the capability of serotonin synthesis through the expression of tryptophan synthetase (O’Mahony et al., 2015).

Psychosomatic comorbidities play a significant role in the generation of symptoms in functional dyspepsia (Ford et al., 2020). Longitudinal studies examining bidirectional effects between the gut and the brain (Koloski et al., 2012, 2016) have revealed that individuals with functional dyspepsia at baseline are more prone to experiencing anxiety or depression during follow-up compared to those without functional dyspepsia, while individuals with anxiety or depression at baseline are more likely to develop functional dyspepsia than those without anxiety or depression. The gut microbiota can engage in bottom-up signaling through the production of metabolites, interacting with the central nervous system via neural pathways or by crossing the intestinal barrier, and modulating neurotransmitter levels, thereby influencing mental wellbeing (Evrensel and Mehmet-Emin, 2015; Barandouzi et al., 2020). In this study, bacterial genera such as *Corynebacterium*, *Clostridium*, and *Streptococcus*, which showed a significant upward trend in two or more studies, exhibiting significant increases in abundance in the duodenum of FD patients, were also found to significantly increase in the gut microbiota of individuals with depression. Conversely, bacterial genera such as *Lactobacillus* and *Prevotella*, which were found to have decreased relative abundance in two or more included studies, were also found to significantly decrease the gut microbiota of individuals with depression (Barandouzi et al., 2020; Park et al., 2023). Furthermore, ingestion of lactobacilli is believed to regulate central GABA receptor expression via the vagus nerve and reduce stress-induced corticosterone as well as anxiety and depression-related behaviors (Bravo et al., 2011).

**Figure 3:**
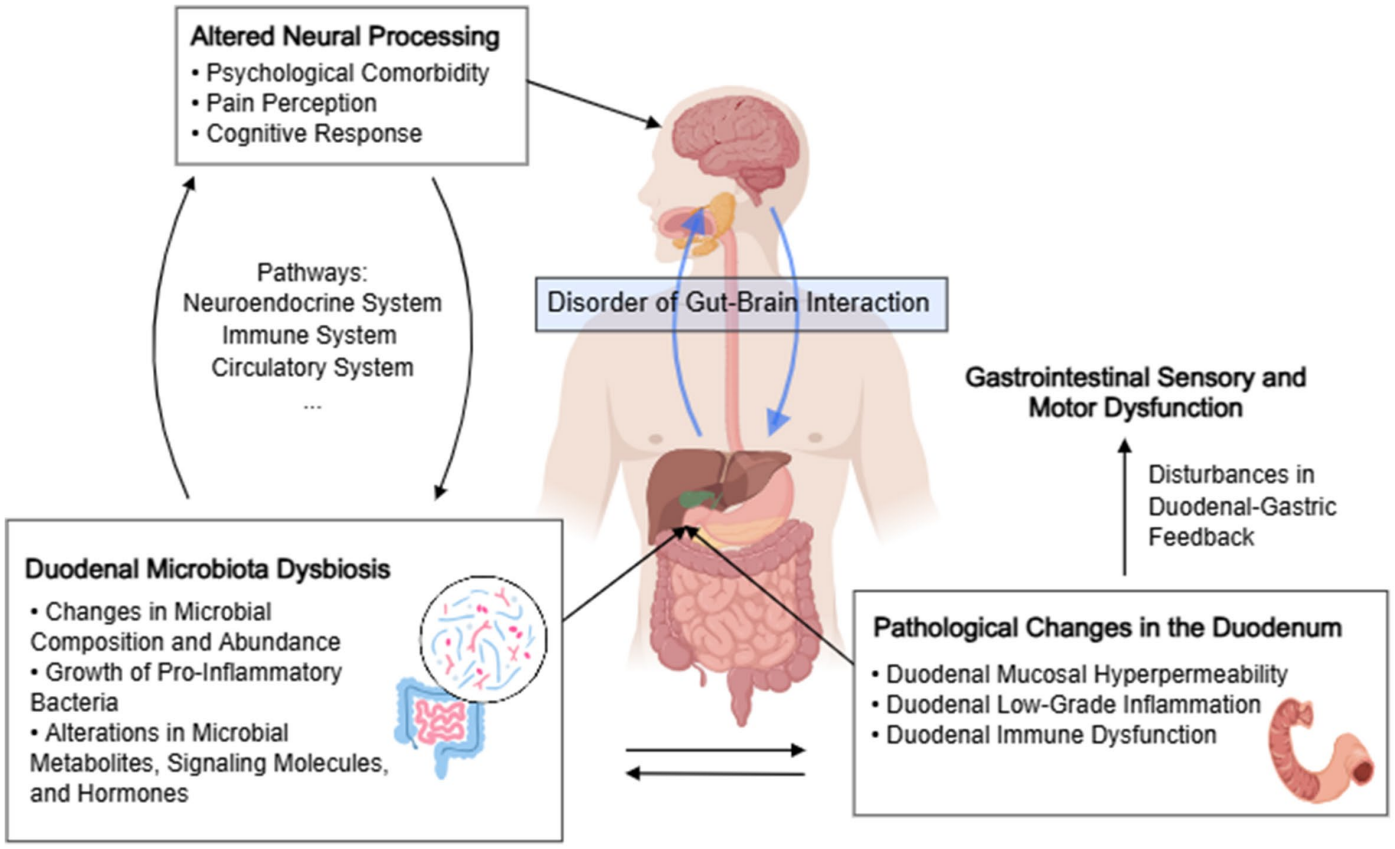
The role of duodenal microbial dysbiosis in disorders of gut–brain interactions in functional dyspepsia. The chart was created by MedPeer.

In summary, the duodenal microbiota plays a role in the gut-brain axis in FD patients. The duodenal microbiota may influence the crucial duodenal functional status implicated in FD pathogenesis through endocrine, neural signaling, and immune-inflammatory responses. Additionally, it participates in the bidirectional brain-gut interactions in FD patients, thus constituting a dysregulated pathogenesis of FD based on the microbiota-gut–brain axis, ultimately leading to the onset of FD (Figure 3).

## 5 Conclusion and outlook

In summary, while the overall diversity of the duodenal microbiota does not significantly differ between FD patients and healthy individuals, certain taxa—including *Selenomonas*, *Streptococcus*, *Prevotella*, and *Staphylococcus*—exhibit notable variations that warrant further investigation. Our study suggests that alterations in the duodenal microbiota may be crucial for the onset and manifestation of FD symptoms. The microbial species significantly altered in this study are known to influence the bidirectional interactions between the brain and gut in FD patients by modulating endocrine, neural signaling, and immune-inflammatory responses, thereby contributing to FD pathogenesis. This highlights the significant role of the duodenal microbiota in the brain-gut interactions of FD patients.

The duodenum is increasingly recognized as pivotal in FD pathogenesis, and the gut microbiota’s role has garnered considerable attention. However, due to technological limitations in previous studies, research on the duodenal microbiota of FD patients remains insufficient. The limited number of studies, small sample sizes, and inconsistent quality of the articles result in contradictory findings, insufficient data for meta-analyses, and challenges in standardized data processing. Consequently, only a general trend of dysbiosis in the duodenal microbiota of FD patients can be discerned, with insufficient evidence to pinpoint key microbial communities.

Future research urgently needs to prioritize higher-quality, larger sample-size studies across diverse populations. Efforts should focus on eliminating bias and incorporating findings from multi-omics studies to further elucidate the characteristics and pathogenic mechanisms of the duodenal microbiota in FD patients. Additionally, attention should be given to confounding factors affecting duodenal microbiota changes in FD patients, such as dietary habits and proton pump inhibitor use. These in-depth studies could provide potential strategic references for the prevention and treatment of FD.

## Data availability statement

The original contributions presented in the study are included in the article/supplementary material, further inquiries can be directed to the corresponding authors.

## Author contributions

XZ: Conceptualization, Data curation, Formal analysis, Methodology, Project administration, Software, Validation, Visualization, Writing – original draft, Writing – review & editing. LC: Data curation, Visualization, Writing – review & editing. TZ: Conceptualization, Methodology, Writing – review & editing. RG: Formal analysis, Software, Validation, Writing – review & editing. QW: Data curation, Project administration, Writing – review & editing. ZZ: Methodology, Supervision, Writing – review & editing. MY: Data curation, Writing – review & editing. WW: Funding acquisition, Methodology, Project administration, Resources, Supervision, Writing – review & editing. XS: Conceptualization, Formal analysis, Funding acquisition, Methodology, Project administration, Resources, Supervision, Visualization, Writing – original draft, Writing – review & editing.
